# Building Reconstruction by Target Based Graph Matching on Incomplete Laser Data: Analysis and Limitations

**DOI:** 10.3390/s90806101

**Published:** 2009-07-31

**Authors:** Sander Oude Elberink, George Vosselman

**Affiliations:** International Institute for Geo-Information Science and Earth Observation, Hengelosestraat 99, P.O. Box 6, 7500 AA Enschede, The Netherlands

**Keywords:** building reconstruction, laser scanner data, target graph matching, incomplete data

## Abstract

With the increasing point densities provided by airborne laser scanner (ALS) data the requirements on derived products also increase. One major application of ALS data is to provide input for 3D city models. Modeling of roof faces, (3D) road and terrain surfaces can partially be done in an automated manner, although many such approaches are still in a development stage. Problems in automatic building reconstruction lie in the dynamic area between assumptions and reality. Not every object in the data appears as the algorithm expects. Challenges are to detect areas that cannot be reconstructed automatically. This paper describes our contribution to the field of building reconstruction by proposing a target based graph matching approach that can handle both complete and incomplete laser data. Match results describe which target objects appear topologically in the data. Complete match results can be reconstructed in an automated manner. Quality parameters store information on how the model fits to the input data and which data has not been used. Areas where laser data only partly matches with target objects are detected automatically. Four datasets are analyzed in order to describe the quality of the automatically reconstructed roofs, and to point out the reasons why segments are left out from the automatic reconstruction. The reasons why these areas are left out include lack of data information and limitations of our initial target objects. Potential improvement to our approach is to include likelihood functions to the existence of topological relations.

## 1. Introduction

In the past few years point the densities of laser point clouds have increased rapidly. Due to the higher pulse rates of laser scanning systems, e.g., see [1], it is possible to acquire large areas with point densities over 10 pts/m^2^. Together with the point densities, the user requirements for 3D buildings have also evolved. With point densities of more than 10 pts/m^2^ the challenge is to reconstruct detailed building parts, besides the general shape of buildings. Problems in detailed automated building reconstruction using airborne laser scanner data are summarized in [2]. The major problem is caused by missing data features, e.g., deflected or absorbed laser pulses, missing laser segments or intersection lines. Although some of the problems caused by incomplete data could be avoided using model driven approaches, hypothesizing the correct topology of the building is still problematic.

Reconstructing buildings automatically is assumed to be timesaving in relation to manual or semi automatic reconstruction. However, a condition that should be fulfilled is that the assumptions used for automated processing are correct for the processed area. Laser data on unwanted objects like trees or cars will have a negative influence on the reconstruction results. In addition, laser data might be missing due to occlusions or lack of returned pulses from non-reflecting surfaces. Automated reconstruction methods should therefore detect and select those areas where assumptions work fine, and at the same time detect areas that need extra attention.

Our first research task is to detect which areas can be reconstructed automatically and which cannot. The methodology for this task is described in Section 3. The second task is then to reconstruct the areas according to the results of the first task. Section 4 deals with the automated reconstruction including its limations, whereas Section 5 explains the reasons why particular segments did not completely match and how these can be resolved.

## 2. Related Work

### 2.1. Building Reconstruction

In the past, many papers have been written on building reconstruction from either photogrammetric data or laser scanner data. A thorough overview of the properties and quality of both acquisition methods is given in [3], where several research groups participated in a comparison test. In this paper we will only focus on laser scanner based approaches. The first efforts dealing with detailed building models were presented a decade ago. Progress has been described in model driven approaches, like in [4] and [5], where the authors fit a selection of predefined models to the data. Data driven approaches often rely solely on the laser data [6], some describe possibilities when integrating laser data with map data [7] and [8]. Reference [9] integrates data and constraints, showing an improvement if constraints are added. Constraints to the data have to ensure that the final model does not take over the irregularity of the input data. Besides the focus on data or model driven, progress has been made in proposing a grammar that describes what the basic elements of roofs and possible roof connections are, like in [10]. The central question “what is a roof?” can only be answered if the grammar is known.

### 2.2. Segmentation

Segmentation of laser data is a process that labels laser points that belong to a certain object, or object type. For several kinds of applications, segmentation can be helpful to process that data. an overview on various segmentation algorithms was presented in [11]. A segmentation based filtering method is described in [12]. The filtering is based on segments, instead of points. For filtering purposes, the authors state that under-segmentation is more harmful to the filtering quality than over-segmentation, as mixtures of terrain and non-terrain points within a segment means either removing too many points or including non-terrain points in a Digital Terrain Model (DTM). In [13] the quality of segmentation is also mentioned as being crucial for the quality of their reconstructed buildings.

### 2.3. Graph Matching for Building Reconstruction

Detecting building shapes by graph matching has been described in [10] and [14]. Based on the detected shapes, buildings are reconstructed. The approach of [14] tends to be more model driven than data driven, as their reconstructed objects are strongly regularized by the model shapes. Incomplete matching results are not taken into further account besides fitting a flat rectangular roof to segments that did not match on a model shape.

## 3. Proposed Methodology

### 3.1. Overview

**Figure 1.**
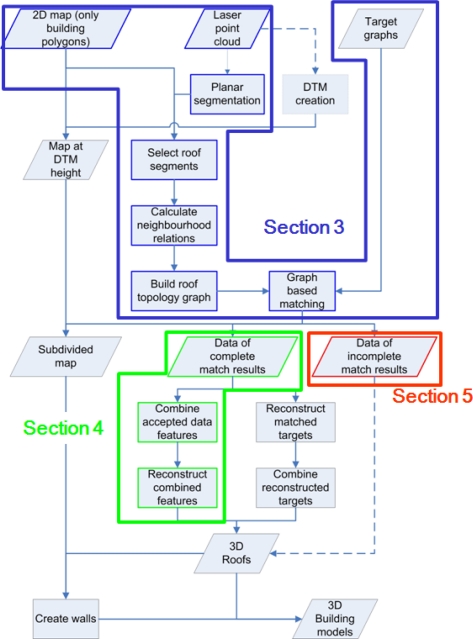
Proposed workflow from input data to 3D building models including paper outline: sections 3 (blue), 4 (green) and 5 (red). Arrows on solid lines indicate automated processes; dashed arrow lines represent semi-automated processes.

Our approach relies on a target based graph matching algorithm, which relates model information with data features. In Figure 1 the workflow of our approach is presented, including the outline of this paper. In order to show the complete algorithm all major steps are given in Figure 1, although some of the steps will not be further discussed in this paper. The basics of this algorithm are described in more detail in [15]. The matching is between a limited number of common roof shapes (targets) and features found in the laser data. Laser data is segmented into planar patches. Patches within, or near, building outlines from map data, are selected for further processing. Step edges and intersection lines implicitly describe topologic relations that can be found between segments. These topological relations between segments are matched with the topology of target objects. Based on these matching results, the outlines of roof faces are reconstructed. Model targets contain information on which constraints can be applied to the corresponding segments and intersection lines. If a complete match is found between parts of the roof topology with a complete target model, these parts can be reconstructed automatically based on the constraints from the model and information from the data. If segments are only matched partly with target objects, there is reason to believe that segments are either missing or superfluous or that the targets do not represent the object.

### 3.2. Data Processing

Airborne laser scanner data was taken as input for our reconstruction approach. The first task is to find laser points that belong to building parts. Reference [13] describes in detail which processing steps are needed to derive roof segments. Its authors describe a hierarchical clustering algorithm for finding seed clusters. These clusters are taken as input for a region growing algorithm where points are added to the cluster if the distance between a plane fitted to the cluster and the point is within certain threshold value. Our assumptions and algorithm are comparable to theirs, as our assumption is also that the majority of roof faces can be described by planar patches. Our surface growing algorithm starts with seed detection in 3D Hough space, followed by a least squares plane fitting through the points in that seed. Nearby laser points are added to the growing surface if points are near that plane. A more detailed description of this method is given in [16]. Segmentation errors include missing segments caused by missing laser pulse returns, over-segmentation due to the fact that the growing radius locally is just too small, under-segmentation caused by the appearance of laser points on two or more objects such that they seem to belong to the same segment. Although our aim is to segment the data as well as possible, we have to accept that segmentation errors do occur. Segments that are located for more than 50% inside a map polygon are assigned to that polygon. Now, for each polygon we continue with the corresponding laser segments. We remove small segments by setting the minimum segment size at 40 laser points. For our datasets of 20 pts/ m^2^, this corresponds with aiming at reconstructing roof faces with a minimum size of 2 m^2^.

### 3.3. Roof Topology Graphs

**Figure 2.**
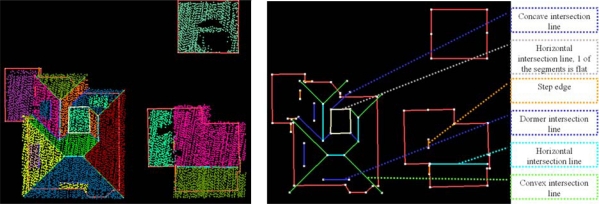
(*Left*) Labeled intersection lines and map outlines, with and without (*right*) segmented laser data.

Generally, the shape of building roofs can be described by the roof faces and the relations between neighboring faces. We take intersection lines and step edges as input to describe relations between faces. These relations are labeled according to their geometry and that of the segments (e.g., same/opposite normal direction, convex/concave, tilted/horizontal). Examples of different labels can be found in Figure 2, where intersection lines are colored by label. Step edges are visualized by an orange line with default length of 1 m. The actual reconstruction of the step edge depends on the reconstruction of the two neighboring faces.

**Figure 3.**
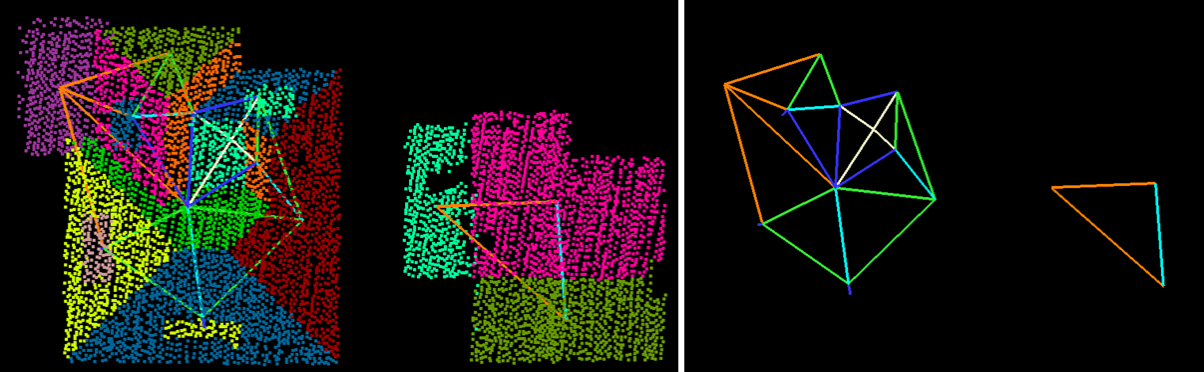
(*Left*) Labeled roof topology graph, with and without (*right*) segmented laser data. Labeling is equal to the labeling in Figure 2.

If a relation exists, we add the existence of this relation to a graph, where segments represent the nodes and the graph edges define the labeled relation between two segments, see Figure 3. Now this labeled roof topology implicitly describes the appearance of the object in the laser data.

### 3.4. Target Based Matching

**Figure 4.**
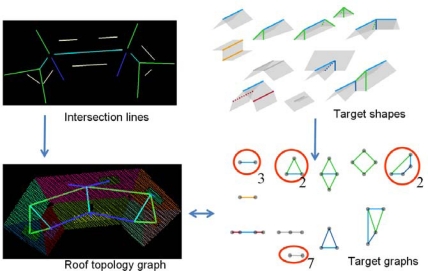
Graph matching algorithm at work: intersection lines and target shapes are represented in a graph structure. Three gable roof types, two half hip shapes, two L-shaped types and seven dormers detected in one building.

We integrate model and data driven approaches by a matching algorithm that relates information from a database to features found in the data. This matching relates the roof segments topology to topological relations between roof faces from a database. Matching between data and model is based on these roof topology graphs and target graphs. As the label of the edges is taken into account, this is called a labeled graph matching algorithm. A more comprehensive description is given in [15]. Using topological descriptions of roof faces for building reconstruction has been described earlier in [17] and [14]. Our approach is an extension of this earlier work, as we include additional attributes to each of the adjacency relations. Besides this, we use the knowledge from the targets database to transfer to the data. This is possible because the matching establishes the link between model and data, so we are able to assign constraints and, if necessary, default values to data features. This is helpful in case the quality of data depends on the type of object, e.g., due to steep slopes at gambrel roofs the quality of derived features such as segments and intersection lines is less than data features from hip roofs. Figure 4 visualizes our approach: intersection lines between segments are represented as edges in the roof topology graph. Graph representations of the target shapes are matched with the roof topology graph.

### 3.5. Matching Results

For each target, multiple match results can be stored per building if that shape appears more than once. Logically, each segment and intersection line can be part of more than one target graph. Results after matching can be input for both model and data driven approaches. The matching performs a filter task: accepted segments and intersection lines are being transferred to the automated roof face reconstruction (Section 4) whereas the segments that did not match completely are transferred to the incomplete match results, which will be discussed later in Section 5.

## 4. Automated Reconstruction of Complete Match Results

### 4.1. Complete Match Results

**Figure 5.**
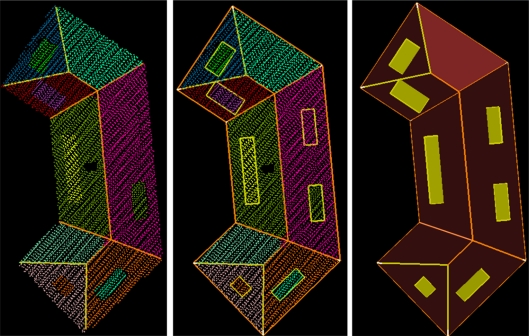
(*Left*) Intersection lines have been extended to intersection points, (*middle*) gutters have been added and dormers reconstructed, (*right*) roof faces represented by closed polygons.

Segments and lines are denoted as accepted if they are part of a complete match between data and target. This means that the structure from (a part of) the roof topology graph exactly corresponds to the target graph. For each segment, all accepted intersection lines are used as input for constructing an outline for the segment. By doing so, we are able to reconstruct any combination of roof structures as long as the intersection line is accepted during the target matching. Important feature of the target based graph matching is that the intersection lines are extended to corner points, e.g., three intersection lines of a half hip roof are extended to one point, and four intersection lines of an L-shaped building have to coincide in one point, see Figure 5. Normally, a segment is partially bounded by intersection lines. Other edges like gutters [in this paper, gutters are (mostly horizontal) edges, created at the lower side of segments, eaves are considered as roof edges between a higher edge (mostly top ridge) and lower edge (mostly gutters)] have to be constructed as that part of the segment does not intersect with another segment. Various ways can be followed to construct these missing face edges. For example, for tilted roofs we can intersect the roof plane with a horizontal plane through the lowest laser point of the segment. For horizontal intersection lines that do not end in an intersection point, e.g., simple gable roofs, eaves are constructed by a perpendicular line to the ridge line, projected to the plane through the segment. Additional information can be in the form of constraints, such as constraining gutter heights to be the same for that specific target shape or for that whole building. Further details on the reconstruction algorithm can be found in [15].

### 4.2. Quality Indicators to Evaluate Processing Steps

Various indicators can be used to describe the quality of the reconstructed models. In this paper we list a limited number of internal quality checks between laser scanner data and 3D model. Rather than to focus on the quality of the end product (to evaluate the 3D model, the assessment would preferably be based on external 3D reference data), we would like to describe how to evaluate the processing steps and assumptions made. For end users as well as for researchers it is of interest to have insight in the consequences of successive reconstruction steps, in order to improve the 3D model or the reconstruction approach itself.

**Figure 6.**
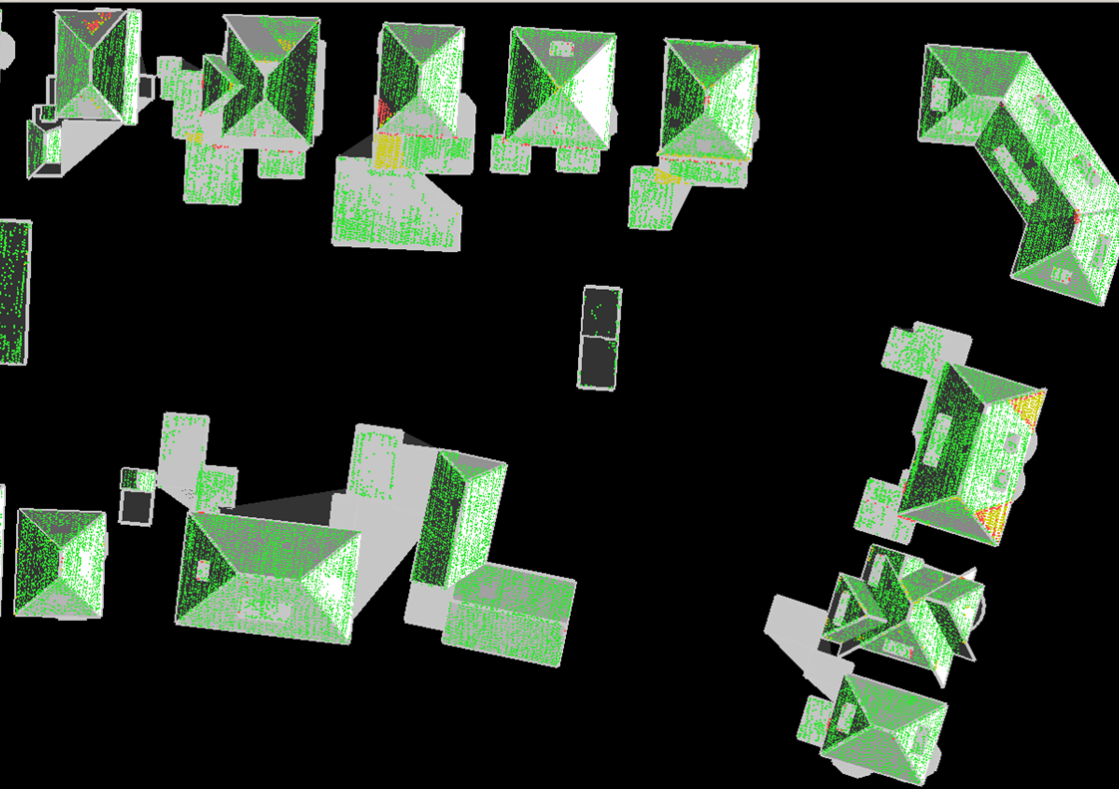
Laser points colored by height difference between laser data and 3D model. Green: less than 20 cm, yellow: less than 50 cm, red: more than 50 cm.

One of the quality checks is the orthogonal height residuals between 3D model and laser points, presented earlier in [13]. This gives a global overview if model and laser data fit to each other, as shown in Figure 6. It is expected that the majority of residuals is colored green (within 20 cm, the acceptance height during planar surface growing in the segmentation step) as the model faces are constructed by least square fitting through the same points. Obviously, large residuals are found on segments that did not match completely, as these laser points were left out from the reconstruction step. Section 5 presents an analysis on these segments. However, we also found large residuals on segments that were part of a complete matching result. It is of interest to elaborate on these data parts as it reveals information on the cases where the data does not fit to the assumptions of the algorithm.

In this section, we focus on residuals of segments that topologically matched completely on target objects. Large residuals on these segments imply that these segments geometrically do not fit to the constraints inherited from the target model, although the segments topologically fit. These situations indicate that errors are made after segmentation, or at least that the laser points do not fit to the assumptions inherited from the target object. If during feature extraction errors are made that have caused a match with an incorrect target, the reconstructed roofs will show height differences on at least a part of the laser points. As each roof face is constructed by fitting a plane through a segment, large residuals are only caused by the fact that laser points are not within the reconstructed roof. Therefore, it is of interest to analyze laser points of segments that were part of a complete match, with residual values above 20 cm. These segments are detected automatically. In Table 1 we have listed statistics of four areas from two cities in the Netherlands. The structure of buildings slightly varies between the data sets, starting with upper class buildings in data set 1 (partly shown in Figure 6), moving to normal sub urban areas in dataset 4 (dataset 3 partly shown in Figure 8).

**Figure 7.**
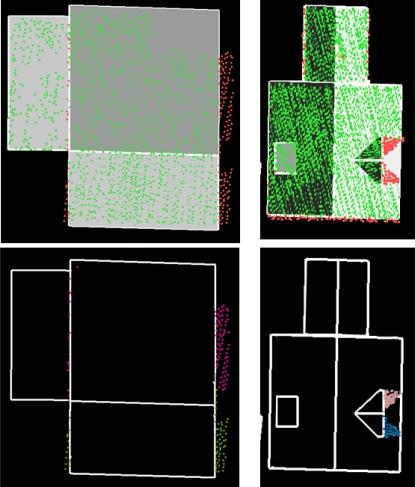
(*Top*) Laser points colored by residual value between laser point and reconstructed roof. (*Bottom*) Segment parts with high residuals due to incorrect assumptions on ridge length (*left*) and target shape (*right*).

The number of affected buildings varies between 5 and 13 percent of the total number of buildings. This variation can be explained by the variations of building parts between the four areas. For example assumptions on equal gutter heights and minimum segment size fit better in one situation than in others. In Figure 7 examples are given of segments that do not exactly fit to the reconstructed roofs, although they were part of a complete match. They show the limitations of our automated reconstruction algorithm. On the left, the intersection line between two gable segments did not completely cover the actual gable ridge. Near the end of the ridge there were no laser points on the gable faces. In fact, a chimney was located at the ridge end. Outlines of these gable faces are constructed perpendicular to the ridgeline. This causes that a part of these segments falls outside the face outlines. On the right, a dormer face is missing due to a missing segment on the hipped part of the dormer. The two remaining segments correspond with a gable shaped dormer. Again, outlines of these segments are constructed perpendicular to the dormer ridgeline, cutting a part from both segments. These situations show that it is possible to detect incorrect assumptions. It is also possible to extend the ridgelines automatically until no more points fall outside the roof face. However, the situation on the right is then ‘solved’ incorrectly, because the correct solution would include the hipped roof face at the location of the missing segment.

Buildings affected by these segments can be shown to the user for an interactive solution or can be used as input data in automated iterative process with changing processing parameters. Various reasons can be assigned to these situations, which make it hard to automatically identify the solution. The fact that these limitations are detected automatically is the first step towards finding solutions for these cases automatically.

So far, we analyzed data that topologically correspond with a target object. However, it is of higher interest to analyze the data that was not included in a complete match and therefore left out from the automatic reconstruction so far. Another important quality parameter therefore is the recording of segments that did not completely match on target objects.

## 5. Analyzing Incomplete Matching Results

### 5.1. Incomplete Matching Results

In the previous section, segments were reconstructed that were part of a complete matching result. Segments and intersection lines that were not part of a complete match have not been reconstructed in the approach described in the previous section. It is of high interest to examine these segments in order to detect incompleteness in the data or the target database. As can be seen in Table 2, this section deals with about 5% of the total number of roof segments, but these affect 19% of the buildings. Remember we removed segments containing less than 40 laser points from the processing, so the table deals with segments on objects larger than about 2 m^2^.

**Figure 8.**
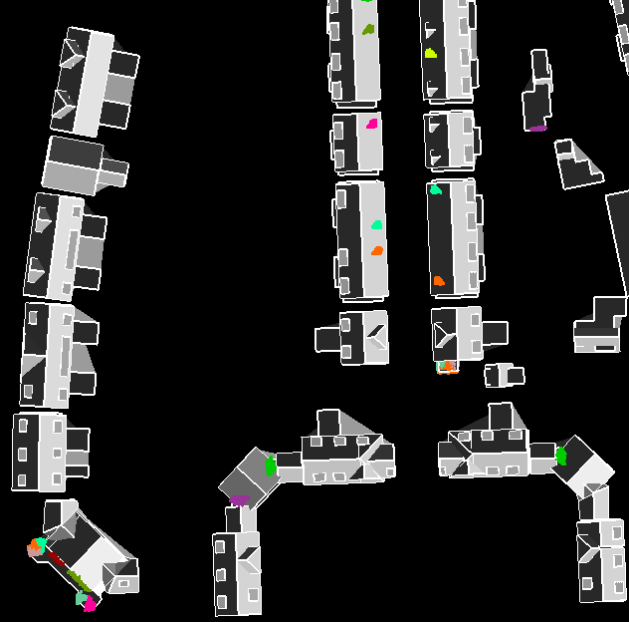
Segments of incomplete match results superimposed on 3D models.

In Figure 8 segments from a small subset are superimposed to the automatically reconstructed building models. It is of interest to analyze these “leftover” segments as these hold important information on the completeness of the match between laser data and model database. First question to be answered is why these segments are not part of a complete target match. As soon as this is known the question is if this should be avoided or solved. In the next section, an overview is given on the reasons why these segments are “leftover”.

### 5.2. Reasons for Incomplete Matches

For the four datasets mentioned earlier in Table 2, we categorized the segments from incomplete matches according to six reasons, which are explained in this section. To each segment we manually assigned one category. Results of this categorization are listed in Table 3. Although the individual numbers depend on local situations in the data and the real world, the table gives a general insight in how the reasons are distributed over the appearances. The first reason deals with segments that are not matched because they are not a roof face. The majority of the examined segments are actually representing a real part of the roof. The reasons that they are not used in the reconstruction can be described by five categories, which are listed as reason 2–6, described in 5.2.2 – 5.2.6.

#### 5.2.1. Non building segments

The first reason discussed here handles segments that actually should be removed for further processing. Planar laser segments can be found on objects that stand close to buildings but are not part of actual building, such as sun marquees and garden furniture. If these non building segments are located (partly) inside the building polygon, they are incorrectly taken as roof segments. The topology between these segments and neighboring roof segments may not match with a target roof model. So, the fact that they are left out from the automatic approach is in this case correct, as they do not represent roof faces. These segments should be removed from further processing for building reconstruction. This group represents about 12% of examined segments.

#### 5.2.2. Absence of neighboring segments

**Figure 9.**
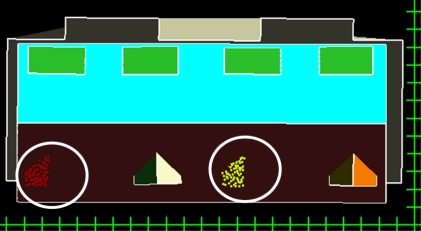
Segments are missing on one of the two sides of gable shaped dormers (white circles); resolution of the scale bar is 1 meter.

The major reason (39%) that a segment is not part of a complete match is that another segment, that would complete a certain target match, is missing. Often, this occurs when the missing segment is on a steep or small object face. For example, many segments in dataset 2 could not be found at one of the two sides of a gable shaped dormer, see the example of Figure 9. As can be seen at the scale bar, the missing segments should represent an object face of about 2 m^2^. For this building six of the eight gable shaped dormer faces could be segmented, and two are missing. Another common problematic case could be found on buildings with gambrel roof shapes where one segment on one of the lower steep roof faces is missing. As a direct result, the segment on the lower steep roof face that actually is found could not be part of a complete match.

#### 5.2.3. Disturbance of topologic relations due to over-segmentation

Another segmentation related reason is the disturbance of topological relations due to over segmentation. Over-segmentation occurs when one (planar) object face is represented by two or more segments. Segmentation errors are made if segmentation parameters locally do not fit to the data. Examples are in situations where platform movements cause large point spacing between two scan lines. If the distance between two scan lines exceeds the growing radius of the segmentation parameters, the segmentation algorithm will not bridge the data gap, splitting up laser points into multiple segments. These segments are treated as individual nodes in the topology graph. This break in the roof topology graph results in a distortion of the matching results.

#### 5.2.4. Absence of topologic relations

Topological relations directly influence the matching results, as they are stored as edges in the roof topology graph. Relations can be absent due to a large distance between two neighboring segments. Large distances can be found near occluded areas and regions with non reflecting surfaces. Examples are found at locations where solar cell collectors are placed near roof edges. The collectors cause local gaps in roof segments and not all intersection lines could be found.

#### 5.2.5. Limitations of the target models

**Figure 10.**
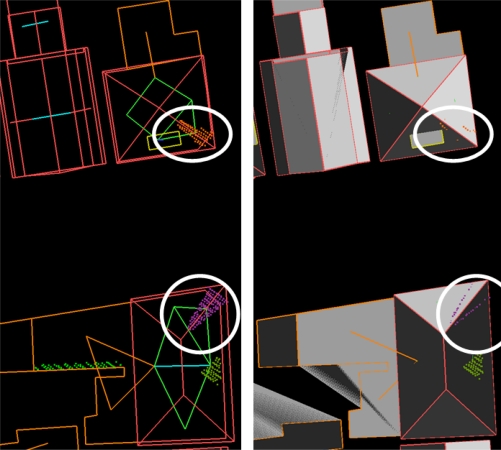
(Left) Segments left over superimposed on reconstructed models, including topological relations of all segments. (Right) As left, but for clarity reasons the reconstructed roof faces have been filled grey.

A roof topology graph might correctly describe a roof shape that is just not in the database. Examples in our dataset can be seen in Figure 10 on a five-sided hip roof and a five-sided pyramid roof. In these cases the limitations of the existing target models cause that the segments on the “fifth” roof face (white circles) are left out from the automatic reconstruction.

#### 5.2.6. Border effects

For several segments on the border of the dataset no neighboring segments or relations could be found. Although this effect is obvious and scientifically not relevant, we mention this category for the sake of completeness of our work.

### 5.3. Discussion on Solving Incomplete Matches

Now the reasons are analyzed, the question is if this should and can be avoided or solved. Can this be avoided by changing the processing parameters? Or should the model database be extended to be able to include these segments such that they are part of a complete match?

First, we discuss the possibility to adapt the segmentation parameters to reduce the number of roof segments that are part of an incomplete match. In order to reduce the errors caused by missing roof segments, the segmentation algorithm should find roof segments at locations where previously the algorithm did not find segments. This can be done by decreasing the minimum of points in a segment in order to be taken as roof segment, or to loosen the acceptance criteria in the growing phase. However, an improvement to one error source might increase the errors from another problem, e.g., disturbance of topological relations, so our suggestion is to apply these changes locally, for example only to buildings that are affected by leftover segments. This can be done in an automated iterative way. The parameters are chosen such that the results that best fit to the user defined quality parameters.

**Figure 11.**
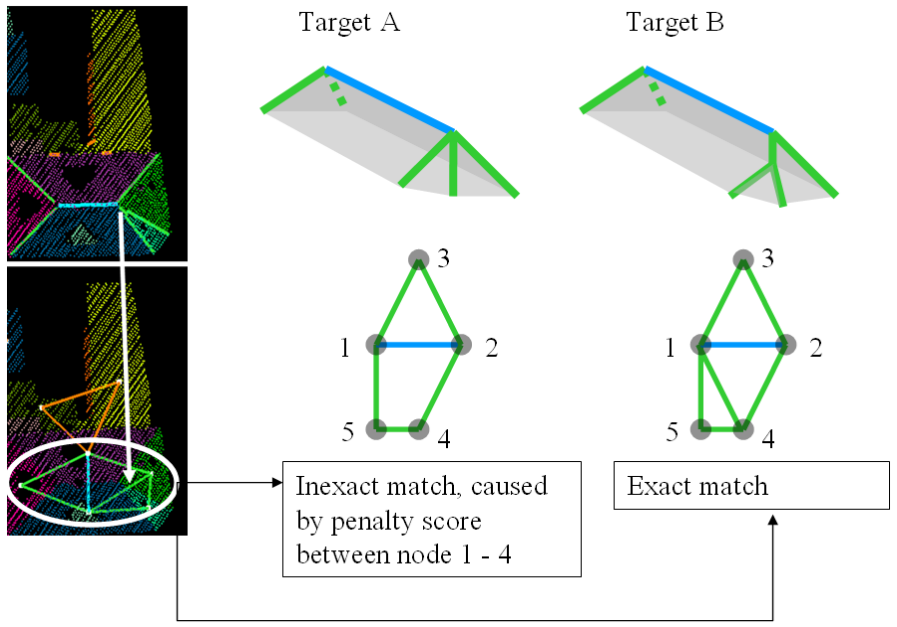
(*Left*) Intersection lines and roof topology graph of a five sided hip roof. (*Middle*) Most likely target A does not exactly match with roof graph. (*Right*) Exact match between data and (unlikely) target B.

The problem of limited number of target models can be avoided by adding new target models to the database. However, we have to keep in mind that the adding more target models that are less common may lead to false matches on other segments that are not part of a building and should be filtered out by the matching. We have to keep in mind that we want to relate object knowledge with features that can be found in the data. To explain the complexity of finding a correct match, we take the situation of a five sided hip roof, as mentioned earlier in Figure 10. On the left in Figure 11 intersection lines and the roof topology graph are shown, on the right in target A and B two target roof shapes are represented as target graphs.

Note that the topological relation indicated by the white arrow, is a result of intersection of two segments that are close to each other. The length of this intersection line can easily exceed the minimum length to be accepted, as is the case in our example. Therefore, from the data side the match with target B is more exact than a match with target A. Match results on target A include a penalty score for the presence of a topological relation in the data that is not in the target between node 1 and 4. However, from our object knowledge we might propose that it is more likely that face 1 and face 4 only meet in one point (target A) instead of sharing a line (target B). In this case our algorithm should ignore the intersection line that caused the penalty score and give preference to a more likely target shape A. This example shows the potential improvement of our target based graph matching by including likelihood estimators to the existence of an object face or a relation between two faces.

## 6. Semi-automatic Processes

As we have seen in the previous section, automated scene interpretation is complicated for regions where data properties are close to threshold values of processing the data. We have explained that the solution to one case might not be correct for another case. In this section we discuss that, at various stages during the processing, the operator can intervene to improve the result. Human intervention is necessary at those places where assumptions in an automatic reconstruction method fail.

Although the aim is to segment the data as good as possible, we do not expect that the segmentation result is perfect. Errors occur if data locally does not fit to the assumptions in the segmentation algorithm. The operator can adjust under- and over-segmentation by splitting or merging segments.

Holes in laser data can be caused by non-reflecting surfaces or due to occlusion by another object. Although classification of data gaps can be highly automated, it is advisable to let the operator check the classification result due to the missing information from the data.

Finding topological relations is an important step in our reconstruction approach as it directly influences the matching results. Based on the matching results the geometry of the segments is reconstructed. Therefore, it is of high interest to check if the topological relations between segments are correct. To support the search for areas of interest, laser points that do not fit to the roof model are detected automatically as explained in Section 4.

## 7. Conclusions

We have presented an approach that can decide whether building segments can be processed automatically or not. This is based on a target based matching algorithm that relates model information with data features. Our automatic reconstruction approach is based on combining intersection lines and step edges that are completely matched with targets from a database. The outer boundaries of each roof face are determined by these intersection lines added by eaves and gutters. The matching algorithm filters out segments and intersection lines did not match completely of a target. About 20% of the buildings are affected by segments that did not completely match with the target graphs. In a few of these cases, this was correct because the segment was not representing a roof face. However, in about 40% of the cases, a neighboring segment that would complete a target match was missing. Adapting processing parameters, such as minimum segment size, might improve the result but it may also disturb other topological relations.

In order to improve our matching algorithm, the likelihood of relations between segments could be included in the attribute list of edges in the roof topology graph. Now, only information on the geometric appearance of the intersection line is given as attribute value to the corresponding graph edge. Future work includes defining likelihood functions for graph edges and analyzing the effect of likelihood attributes.

## Figures and Tables

**Table 1.** Statistics on segments that do not exactly fit on reconstructed roofs.

**Area ID**	**1**	**2**	**3**	**4**	**Total**
# buildings	61	191	226	250	728
# laser points in roof segments	176k	686k	598k	161k	1621k
# laser points with residual > 20 cm	3.1k (1.8%)	17.7k (2.6%)	11.9k (2.0%)	5.8k (3.6%)	38.5k (2.4%)
# segments with more than 20 points with residual>20 cm	4	35	26	21	86
# affected buildings	3 (5%)	24 (13%)	18 (8%)	13 (5%)	58 (8%)

**Table 2.** Statistics on segments of incomplete match results.

**Area ID**	**1**	**2**	**3**	**4**	**Total**
# buildings	61	191	226	250	728
# roof segments	462	1489	1447	798	4196
# roof segments not in complete match (%)	18 (4%)	64 (4%)	46 (3%)	71 (9%)	199 (5%)
# affected buildings	12 (20%)	37 (19%)	35 (15%)	55 (22%)	139 (19%)

**Table 3.** Reasons for segments being part of an incomplete match result.

**Area ID**	**1**	**2**	**3**	**4**	**Total**
# of segments leftover	18	64	46	71	199
1. Non building segment	3 (12%)	9 (14%)	9 (20%)	3 (4%)	24 (12%)
2. Absence of neighboring segments	7 (39%)	28 (44%)	16 (35%)	27 (38%)	78 (39%)
3. Disturbance of neighborhood relations due to over-segmentation	3 (17%)	5 (8%)	4 (9%)	3 (4%)	15 (8%)
4. Absence of neighborhood relations	3 (17%)	10 (16%)	4 (9%)	20 (28%)	37 (19%)
5. Target shape not in database	2 (11%)	7 (11%)	3 (7%)	14 (20%)	26 (13%)
6. Segment on border of dataset	0 (0%)	5 (8%)	10 (22%)	4 (6%)	19 (10%)
